# Spontaneous Hepatic Rupture with Intraperitoneal Hemorrhage without Underlying Etiology: A Report of Two Cases

**DOI:** 10.5402/2011/610747

**Published:** 2011-04-17

**Authors:** Kristin Klein, A. M. James Shapiro

**Affiliations:** Department of Surgery, University of Alberta, 2000 College Plaza, 8215 112th Street, Edmonton, AB, Canada T6G 2C8

## Abstract

Spontaneous hepatic rupture is a rare event most often occurring in association with underlying liver disease or pregnancy. We report two unusual cases of hepatic rupture without any identifiable pathology, trauma, or comorbid conditions.

## 1. Introduction

Massive hemoperitoneum caused by spontaneous rupture of the liver is a rare event, most often associated with benign and malignant hepatic neoplasms [1]. There have been several reports in the literature of liver hemorrhage related to pregnancy [2], and other more rare conditions such as coagulation disturbances [3], connective tissue disease [4], and hypereosinophilic syndrome [5]. However, hepatic rupture in the absence of underlying pathology is an extremely rare occurrence. We describe two cases of patients presenting with liver rupture requiring hepatic resection with no clear etiologic cause and without history of abdominal trauma.

## 2. Case 1

**Figure 1:**
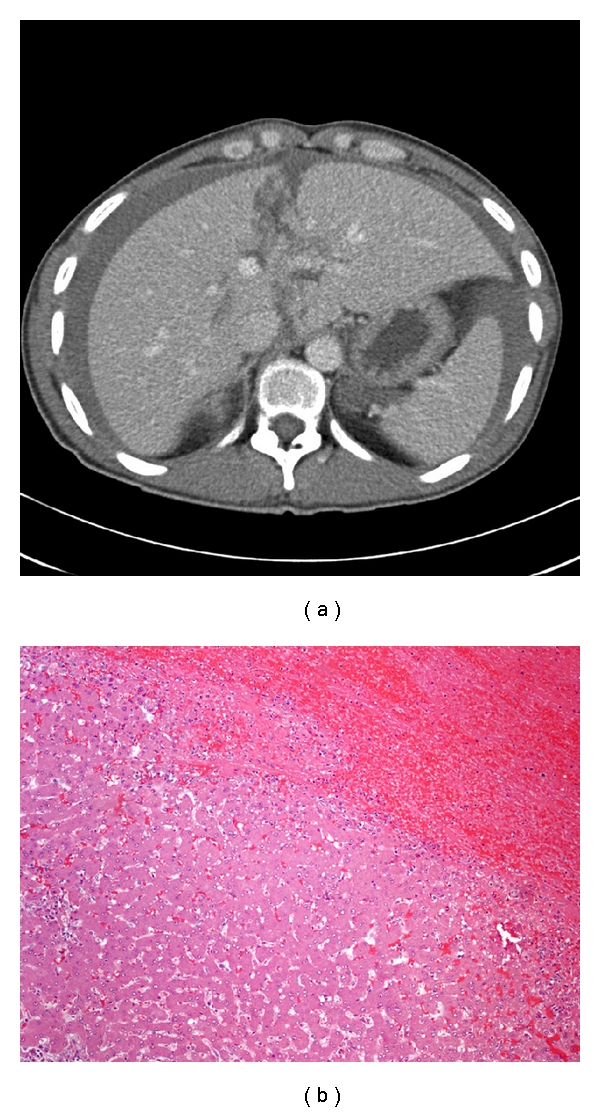
(a) Contrast-enhanced abdominal CT revealing hemoperitoneum. (b) Normal hepatic architecture adjacent to hematoma (H&E, 10x).

A 29-year-old male, previously well, presented to the Emergency department of a peripheral hospital with a two-day history of abdominal pain and emesis. A CT scan of the abdomen revealed hemoperitoneum (Figure 1(a)). The patient was then transferred to a tertiary care hospital with a diagnosis of a ruptured liver tumor despite no history of trauma. 

On examination, the patient was hypotensive, tachycardic, and had a firm, tender abdomen. Hemoglobin was 96 on presentation for which he received 2 units of packed red blood cells. He had mild elevations in his liver enzymes, with AST 257, ALT 418, and had negative hepatitis serology. The CT scan of the abdomen revealed a large amount of free fluid in the abdomen, consistent with hemorrhage. A hypodense liver lesion was seen, and the remainder of abdomen was normal. The working diagnosis based on the images was a spontaneous hemorrhage of a hepatic lesion whose differential diagnoses included hemangioma, adenoma, hepatocellular carcinoma, angiomyolipoma, or metastasis. 

The patient was taken to the operating room emergently for laparotomy and possible liver resection. After the initial right subcostal with midline extension incision was made, approximately 1.5 L of frank hemoperitoneum was evacuated. The patient was found to have a space-occupying lesion in segments II, III, Iva, and IVb of the liver. Laparotomy revealed no other lesions in the liver or bowel. As the lesion was presumed to be a tumor, a left hepatic lobectomy was carried out with grossly negative margins. The patient was stable throughout surgery, and the specimen was sent to pathology for further assessment. 

On pathology, the liver was found to have significant interstitial and subcapsular hemorrhage (Figure 1(b)). However, no intrinsic hepatic disease was noted, and there was no evidence of a benign or malignant neoplastic process. No source for the hemorrhage was identified. Importantly, there was no evidence of vasculitis or other connective tissue diseases seen.

The patient was discharged from hospital in stable condition on postoperative day six with a final diagnosis of a spontaneous liver hemorrhage, and in subsequent followup over two years later, remains well with no residual concerns.

## 3. Case 2

A 46-year-old female presented to a community hospital emergency department with a one-day history of abdominal pain radiating to the right shoulder and light-headedness. Past medical history was significant for Myasthenia Gravis. The patient was hemodynamically stable, and physical examination was unremarkable other than abdominal tenderness to the right upper quadrant. Laboratory tests were significant for an elevation in ALT to 337 and AST to 140 and a positive D-dimer of 1.17. Based on nonspecific symptoms and the positive D-dimer, a CT chest was ordered to rule out pulmonary embolism. 

**Figure 2:**
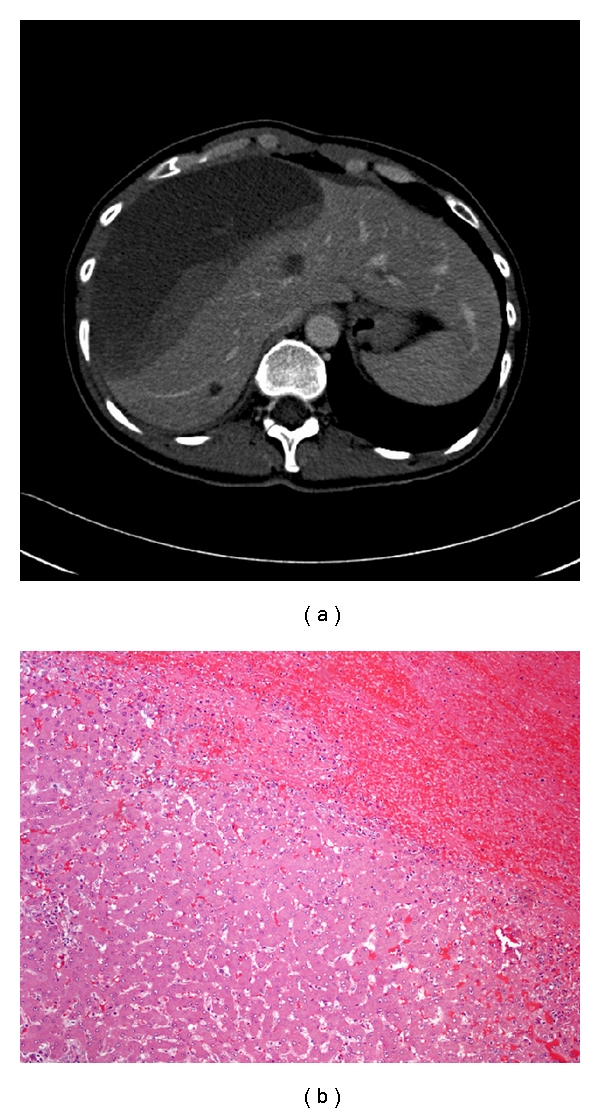
(a) Contrast-enhanced abdominal CT showing massive hemoperitoneum. (b) Normal hepatic architecture with sinusoidal dilatation, adjacent to hematoma (H&E, 10x).

CT of the chest showed no evidence of thromboembolism however, due to some elevation of the right hemidiaphragm, a portal venous scan through the abdomen was undertaken. This examination revealed a very large heterogeneous low-density subcapsular collection, involving almost the entire right lobe of liver measuring 16 × 7 × 16 cm (Figure 2(a)). At the inferior aspect of the lesion, there was an area identified of slightly greater enhancement. There were multiple additional low-density lesions identified throughout the liver parenchyma in keeping with benign cysts. Upon the completion of the study, the working diagnosis was a ruptured hepatic adenoma. 

Based on the results of the CT and progressive tachycardia, the patient was booked for an emergency laparotomy. On examination the entire right side of the liver was grossly distended with subcapsular blood. A right hepatic lobectomy with partial liver resection of segments IVa and IVb was performed without complication. On pathology the specimen was found to contain an extensive subcapsular hematoma with no evidence of neoplastic disease or other abnormal process (Figure 2(b)). Again, no evidence of vasculitis or other connective tissue disease was identified in the pathology specimens. The patient recovered well from the surgery and was discharged home on postoperative day 7 with a final diagnosis of a spontaneous liver hemorrhage. In followup one year later, the patient remains well with no residual abnormalities.

## 4. Discussion

As first described by Abercrombie in 1844 as a complication of pregnancy, spontaneous liver rupture remains a rare event [6]. There have been over 100 cases reported in the literature, with the majority being associated with pregnancy-induced-hypertension as well as primary and metastatic liver tumors [7]. 

The treatment for spontaneous liver hemorrhage in an unstable patient can include hepatic artery embolization, lobectomy, and packing. In both of our patients, the decision was made to complete a hepatic resection due to hemodynamic instability and the inability to exclude malignancy based on preoperative imaging. Diagnosis of liver rupture can be made on the basis of history and physical exam, basic laboratory tests, and abdominal imaging. However, the etiology of the liver rupture may be more difficult to diagnose. Potentially identifiable causes of hepatic rupture include trauma, unrecognized coagulopathy, collagen vascular disease, and rarely microaneurysm formation from inflammatory processes. However, in these two cases, we were unable to uncover underlying liver pathology or inflammatory processes that could have led to hepatic rupture. No evidence of inflammation or microaneurysms were seen on either CT scans. It is possible that a more dedicated angiogram might have uncovered a microaneurysmal process, but this is felt to be unlikely based on the thorough pathological examinations. Subclinical trauma remains a remote possibility, but in both cases, the subjects and their families were adamant that there was no antecedent abdominal trauma or deceleration-type injuries. However, as demonstrated by both of these cases, spontaneous liver rupture does not necessarily infer definable underlying liver pathology.
